# A risk prediction model for HPAI outbreaks in Kuwait

**DOI:** 10.3389/fvets.2026.1843962

**Published:** 2026-05-28

**Authors:** Musab Alshatti, Ali Al-Hemoud, Ahmad Othman, Hanan Al-Khalaifah

**Affiliations:** 1 Systems and Software Development, Science and Technology Division, Kuwait Institute for Scientific Research, Safat, Kuwait; 2 Environment and Life Sciences Research Center, Kuwait Institute for Scientific Research, Safat, Kuwait

**Keywords:** HPAI, Kuwait, logistic regression, machine learning, risk mapping

## Abstract

**Introduction:**

Highly pathogenic avian influenza (HPAI) outbreaks pose significant threats to animal and human health. Risk assessment and prediction modelling are essential for improving disease control and enabling timely intervention strategies.

**Methods:**

HPAI outbreak data collected over 16 years (2005–2020) were integrated with meteorological data, wild bird nest proximity, and confirmed outbreak locations. Logistic regression and machine learning tools were used to predict HPAI outbreaks.

**Results:**

The model demonstrated strong predictive performance, achieving a balanced accuracy score of 0.79 and a ROC AUC of 0.83. The study identified Kuwait metropolitan City and the coastline as the most vulnerable locations for HPAI outbreaks.

**Discussion:**

This study provides a foundation for developing spatially targeted HPAI control strategies in Kuwait. Risk prediction and mapping can support early response efforts and surveillance prioritization during high-risk periods.

## 1 Introduction

Avian influenza disease outbreaks are a global animal health concern. Understanding the avian immune response, particularly the role of cytokines as key regulators of both innate and adaptive immunity, is essential for developing effective strategies against highly pathogenic avian diseases (1). The development of an early detection system for zoonotic diseases is essential. Disease outbreak research has become a central component of modern epidemiology, particularly with the increasing integration of data-driven approaches for early detection and response. Recent studies highlight that infectious disease outbreaks are strongly influenced by a combination of environmental, climatic, and socio-economic drivers, which can be effectively captured using advanced computational methods. In particular, artificial intelligence (AI) and machine learning (ML) techniques have been widely applied to improve outbreak forecasting by integrating heterogeneous datasets, including meteorological variables, population mobility, and health surveillance records (2). Consistent with this, recent research demonstrates the potential of data-driven modeling approaches to enhance outbreak prediction and risk assessment. AI-based models have been shown to improve decision-making processes by enabling more accurate predictions compared to traditional time-series methods, particularly when incorporating human mobility patterns alongside conventional epidemiological factors (3). Furthermore, advances in model interpretability, such as the use of Shapley Additive exPlanations (SHAP), allow for a better understanding of the contribution of individual variables to model outcomes, thereby increasing transparency and trust in predictive models (4).

Most risk prediction models depend on historical data to recognize patterns of outbreaks (5). The World Organization for Animal Health (WOAH, formerly OIE) was among the first organizations to realize the importance of developing a worldwide database for animal diseases. WOAH has been collecting information on animal health diseases since 1993 and collects qualitative and quantitative data on selected diseases of wild animals. Since 2012, the World Animal Health Information System (WAHIS) provided a public WAHIS-Wild Interface (6).

There are a limited number of studies that developed risk prediction models for HPAI outbreaks. An early warning system to monitor and track five animal disease outbreaks [highly pathogenic avian influenza (HPAI), foot and mouth disease (FMD), glanders, lumpy skin disease (LSD), and Middle East respiratory syndrome coronavirus (MERS-CoV)] was recently developed (7). The authors used WAHIS data and Tableau to visualize disease spread patterns across Kuwait to effectively identify animal threats and disease outbreaks. Continuous monitoring of potentially hazardous viruses, including avian influenza viruses, avian coronaviruses, and SARS-CoV-like viruses, is critically important to safeguard both humans and animals from zoonotic diseases that could trigger global pandemics (8). A threshold system was developed for HPAI and low pathogenic avian influenza (LPAI) outbreaks in the Netherlands (9). The threshold system was based on collected data from 30 Dutch farms and included daily mortality and egg production rates; the early detection system was triggered if, for two consecutive days, the mortality rate was higher or the egg production was lower than the calculated threshold. Another study modeled HPAI disease outbreaks using several variables (chicken density, domestic waterfowl population density, proportion of land covered by surface water, cropping intensity, elevation, and human population density) (10). The authors used bootstrapped logistic regression and boosted regression trees (BRT) with cross-validation to identify the weight of each variable and map the distribution of HPAI risk as early detection of an occurring outbreak. Their results showed that the HPAI H5N1 clinical disease outbreak occurrence in domestic poultry was mostly related to chicken density, elevation, and human population density. On the other hand, HPAI H5N1 infection identified by risk-based surveillance was associated with domestic waterfowl density, human population density, and the proportion of land covered by surface water. The potential risk of HPAI due to the flow of live poultry in live bird markets and traders in northern Vietnam was studied (11). The results showed that live poultry traders play an important role in the transmission of the HPAI H5N1 outbreak. Several risk factors that lead to the spread of the HPAI virus in poultry in West Java Province, Indonesia were studied (12). Risk factors included poultry density, human density, road density, and percentage of paddy fields. The analysis was based on Poisson regression using generalized linear modeling and generalized estimating equations. Their results showed that both poultry density and road density had a statistically significant correlation with the number of HPAI outbreaks in poultry. The number of poultry outbreaks had a negative association with poultry density (29% effect) and a positive association with road density (67% effect). The number of human cases was significantly associated with the number of poultry outbreaks (34% effect).

Due to the limited number of studies on HPAI outbreak risk prediction, this study aimed to develop a risk prediction model and mapping for HPAI outbreaks in Kuwait using logistic regression and practical web-based interactive tools. The poultry industry in Kuwait is a vital agricultural sector, and protecting it from HPAI outbreaks requires robust early detection systems that account for local production characteristics and environmental conditions (13). Early detection of HPAI is vital to control the transmission of zoonotic diseases. Zoonotic disease transmission can be minimized by inducing a risk prediction model using spontaneous detection and reporting of clinical signs at the start of disease outbreaks.

## 2 Methods

### 2.1 Outbreaks and wild bird data

HPAI-reported outbreaks were collected for 16 years (2005–2020) from the WOAH. The data were classified by: (a) Geographical Coordinates: Latitude and longitude of the outbreak location, (b) Administrative Region: The governorate or administrative area affected, (c) Location: Specific towns or areas within the administrative region, (d) Serotype: Identification of the avian influenza virus strain (e.g., H5N8 HPAI, H5N1 HPAI), (e) Species Affected: Classification of birds impacted (domestic, wild), (f) Birds at Risk: Total number of birds susceptible to infection, (g) Cases: Number of confirmed infected birds, (h) Deaths: Number of bird fatalities, (i) Destroyed: Number of birds culled to prevent further spread.

Wild bird data were also collected to gather information about various bird species, their habitats, and nesting patterns across different months. Wild bird data classification was: (a) Family Name: The broader taxonomic group to which the bird species belongs, (b) Scientific Name: The binomial scientific name (genus and species) for the bird, (c) Local Name: Common names for the bird species, potentially in different languages or regions, (d) Residence Status: Indicates whether the bird is a resident (present year-round), a winter visitor, a summer visitor, or migratory in the region, (e) Habitat Types: Describes the typical environments or ecosystems where the bird species is found (e.g., wetlands, forests, grasslands), (f) Month (JAN to DEC): Monthly columns contain presence indicators, likely numerical values (0 to 3) representing the abundance or likelihood of observing the species during that specific month, (g) Geometry: Geographical coordinates, stored as “MULTIPOINT” data, indicating the specific locations where the bird species have been recorded or observed.

### 2.2 Weather data

The prediction model integrated meteorological data obtained from the Kuwait Meteorological Department extracted from a total of 18 stations across Kuwait. This data spans multiple years, offering insights into long-term weather patterns and essential climate metrics. These stations are geographically diverse, covering coastal areas like Bubiyan Island and urban districts such as Kuwait City. The dataset includes: (a) Station Name: The name of the weather station, (b) Geographical Coordinates: Latitude and longitude of the weather station, (c) Average Temperature: The mean temperature recorded at the station (e.g., 25.1 °C to 28.3 °C), (d) Relative Humidity: The average relative humidity, showing significant variation across locations, (e) Wind Speed: The average wind speed, (f) Annual Rainfall: The total annual precipitation. Ordinary Kriging techniques were applied to interpolate meteorological data onto a regular grid within the study area using the GSTools and PyKrige libraries, leading to the creation of four feature layers (temperature, relative humidity, annual rainfall, and wind speed).

To predict the risk of HPAI outbreaks and develop a risk map, we used the following machine learning programming tools: (1) Jupyter Lab as a web-based interactive development environment for data and coding; (2) Python 3.8 was used as the programming language; (3) Statsmodels to conduct statistical tests (logistic regression); (4) Pandas on top of the Python programming language for data analysis and manipulation; (5) Matplotlib for interactive visualization and risk mapping.

### 2.3 Pseudo-absence design and sampling strategy

The sampling frame was deliberately restricted to areas considered epidemiologically relevant to HPAI transmission in Kuwait. Urban environments were included due to the presence of high-risk interfaces such as the Friday live bird market in Kuwait City and backyard poultry holdings, which are recognized as potential introduction and amplification nodes. Farm-proximate areas were also included, as commercial poultry production is spatially concentrated in regions such as Al-Wafra and Sulaibiya, where gradients in biosecurity and interactions between wild and domestic birds create heterogeneous but plausible exposure conditions. In contrast, areas deemed epidemiologically implausible for HPAI transmission were excluded from the sampling frame, including (i) open desert regions located >10 km from any poultry holding or market, (ii) offshore islands without poultry activity, and (iii) restricted military zones lacking agricultural activity. This restriction follows the principle that pseudo-absences should be drawn from the accessible population of locations that could plausibly experience an outbreak, rather than from the entire geographic extent, thereby improving epidemiological comparability between presence and pseudo-absence locations and reducing bias arising from structurally unsuitable environments.

Pseudo-absence points were allocated equally between urban and farm areas to ensure balanced representation of the two dominant epidemiological contexts relevant to HPAI transmission. Urban areas capture anthropogenic mixing interfaces, including live bird markets and backyard systems, whereas farm areas represent commercial poultry production environments. Given the limited number of observed outbreaks, this stratified design was adopted to prevent over-representation of a single landscape type and to enable the model to distinguish between these key risk settings. However, this allocation is heuristic and not proportional to the true population at risk, and this limitation is acknowledged.

To evaluate the robustness of the pseudo-absence design, repeated random sampling was conducted (*n* = 100 iterations) under the same spatial constraints (5 km exclusion buffer and urban–farm stratification). Model performance metrics (balanced accuracy and ROC-AUC) and parameter estimates were compared across iterations, demonstrating minimal variability and indicating that model outcomes were not sensitive to a single random draw.

To further assess sensitivity to pseudo-absence specification, four alternative sampling strategies were implemented and compared. Target-group background sampling was used to account for shared surveillance bias by drawing pseudo-absences from locations where other notifiable diseases were reported, following established approaches for bias correction (14). Environmentally stratified sampling was applied to ensure that pseudo-absences occupy similar environmental space as presence locations, thereby reducing spurious model discrimination based solely on environmental gradients (15). Distance-based exclusion strategies were evaluated using multiple buffer thresholds, consistent with recommendations that pseudo-absence distance influences model performance (16). Finally, a uniform random background sampling approach was used as a baseline, despite known limitations associated with uninformed pseudo-absence selection (17).

Across all alternative strategies, model coefficients, predictor importance, and spatial risk patterns remained consistent, with high-risk areas persistently identified in metropolitan Kuwait and the Wafra farming region. While minor differences in model performance were observed, these did not alter the overall interpretation, indicating that the study conclusions are robust to the choice of pseudo-absence design.

### 2.4 Negative sample and grid point generation

A critical step in our spatial analysis involved the generation of a negative sample dataset. This was achieved using a custom Python script “generate_negative_sample.py.” The script systematically creates random geographical points within predefined areas of Kuwait, specifically focusing on urban built-up zones and regions in the vicinity of poultry farms. Geospatial data and random sampling techniques were employed to ensure that all generated points strictly adhered to the specified boundaries, thereby preserving ecological validity. These negative samples function as control data, providing a necessary baseline for comparison with positive samples (i.e., known avian influenza outbreak locations). This comparative approach facilitates the identification of spatial patterns, assessment of risk factors, and validation of predictive models for avian influenza spread.

Moreover, a grid of evenly spaced geographical points across Kuwait was generated using the “generate_grid_points.py” script. This involved reading Kuwait’s governorate Shapefile and reprojecting it to EPSG:31901 for accurate meter-based calculations. The bounding box of Kuwait was determined, buffered, and then points were iteratively generated at 5,000-m intervals. The resulting GeoDataFrame was exported as “grid_points.csv,” providing a structured framework for subsequent spatial analyses and modeling.

### 2.5 Feature engineering, combination generation, and selection

In this study, each grid point represents a single spatial observation (unit of analysis). The response variable was defined as a binary outcome, where 1 indicates the presence of an HPAI outbreak and 0 indicates its absence. Positive cases correspond to confirmed outbreak locations, while negative cases were generated as pseudo-absence points outside a 5 km buffer from known outbreaks.

Predictor variables were selected based on epidemiological relevance and the availability of spatially consistent data across the study period (2005–2020). The feature set was restricted to proximity-based indicators (wild bird nests and historical outbreaks) and meteorological variables, which could be uniformly derived at the national scale. Anthropogenic and production-related variables (e.g., poultry density, market locations, and movement networks) were considered but not included due to limited availability of harmonized, high-resolution datasets.

For each grid point, predictor variables were systematically calculated using spatial queries, including distance-based measures and buffer-based counts (e.g., proximity to outbreaks and wild bird nests), along with associated meteorological attributes. This approach ensures that both the response variable and all covariates are consistently defined at the same spatial resolution for model training and prediction.

The generated risk map represents a continuous probability surface, with values ranging from 0 to 1, rather than a binary classification of outbreak versus non-outbreak areas. High-risk (red) regions indicate relatively higher predicted probabilities of HPAI occurrence, not definitive outbreak locations. Therefore, the map is intended to support surveillance prioritization and risk stratification rather than direct implementation of uniform control measures across all highlighted areas.

Spatial and meteorological features were engineered using the “features_calculation.py” script. This script integrated several geospatial datasets: weather station data, historical avian influenza (AI) outbreak locations, generated negative samples, wild bird nest sites, and a pre-generated grid of points. All datasets were converted to GeoDataFrames and re-projected to EPSG: 31901 for consistent spatial analysis. For each grid point, the following spatial features were calculated: (a) Number of historical AI outbreaks within 10 km, 20 km, and 30 km, (b) Number of wild bird nests within 10 km, 20 km, and 30 km, (c) Distance to the nearest wild bird nest, (d) Distance to the nearest historical AI outbreak. A logarithmic transformation was applied to distance features for normalization. Meteorological data were incorporated by associating each grid point with its closest weather station and extracting average temperature, relative humidity, wind speed, and annual rainfall. The script generated labeled datasets for model training and prediction, and a refined grid dataset for risk map generation, both exported as CSV files.

Feature combination generation used the “features_combinations.py” script to systematically generate all possible combinations of feature columns from the labeled dataset. This script read the dataset, excluded the “geometry” column, and applied combinatorial logic to create subsets of the available features. These feature combinations were then exported to a CSV file, providing a comprehensive set of feature groups for subsequent model training and evaluation. This step allowed for a robust exploration of feature interactions and selection of optimal feature sets for predictive modeling.

Feature selection utilized the script “features_selection.py” to identify optimal feature subsets for predicting avian influenza risk. This involved loading labeled datasets, standardizing feature values, and randomizing the data to minimize bias. Logistic regression models were trained and evaluated using cross-validation across various feature combinations. Model performance was assessed using balanced accuracy, precision, recall, F1 score, negative log loss, and ROC AUC. The top-performing feature sets, based on these metrics, were then recorded for subsequent analysis. This systematic approach ensures robust model development and highlights key factors influencing avian influenza risk.

**Figure 1:**
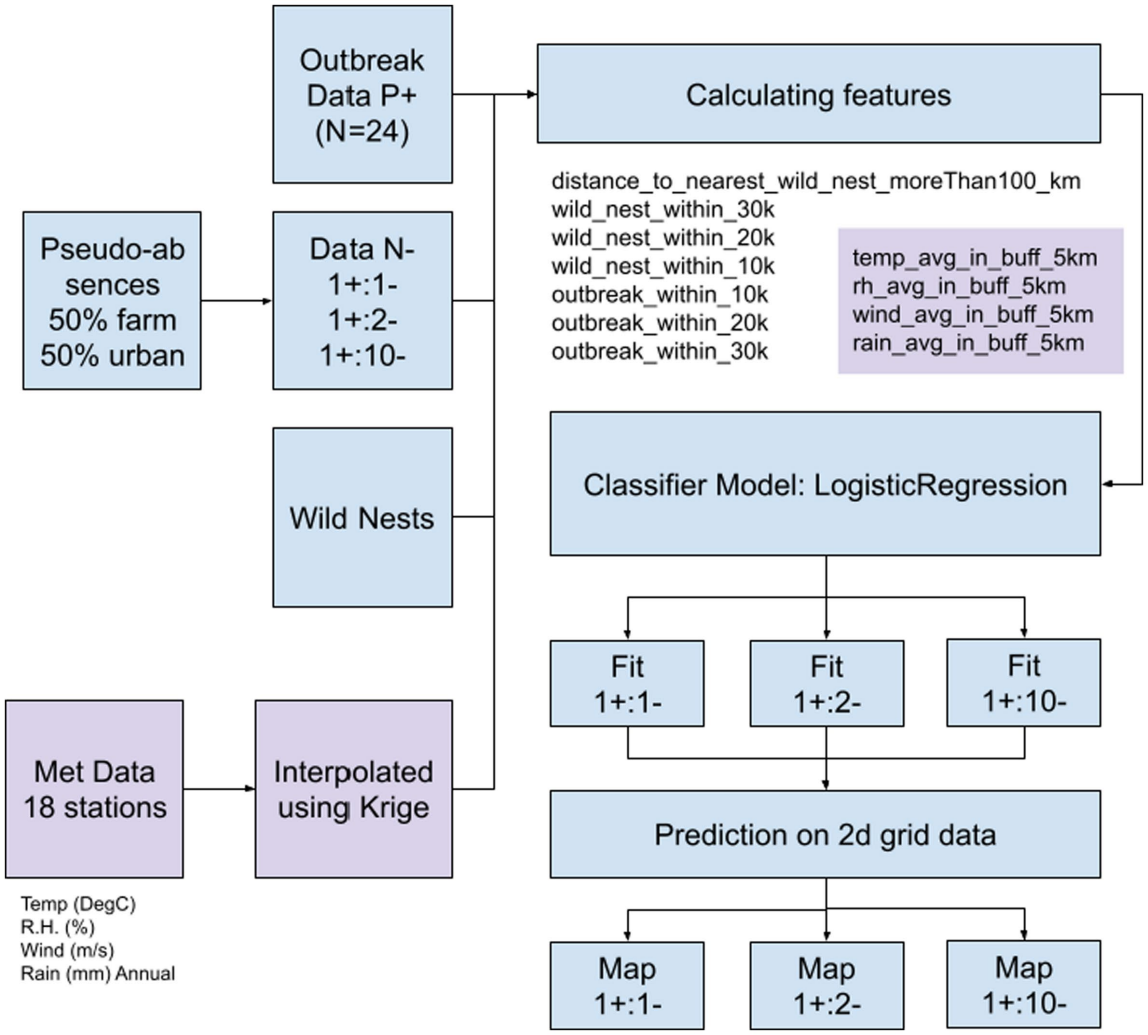
Overview of the model used to predict HPAI outbreaks.

**Figure 2:**
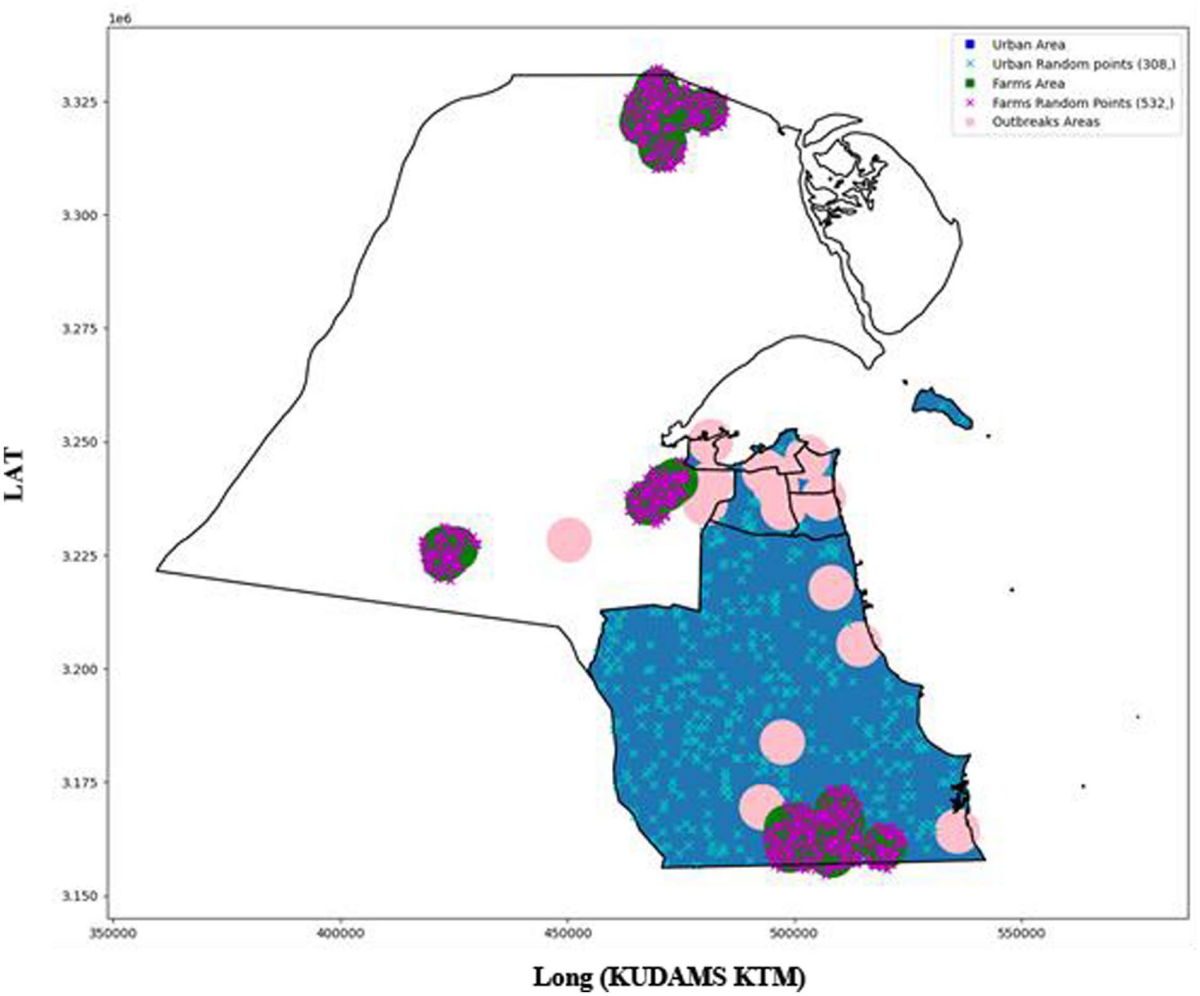
Sampling points used in the regression model to predict HPAI outbreaks.

A Logistic regression model was used to study HPAI outbreaks (Figure 1). We processed the calculated features from the meteorological data, buffers and distance of wild nests, and positive and negative instances of avian flu outbreaks. The calculated features were standardized using the StandardScaler from sklearn. The model was evaluated using a 5-fold cross-validation, with multiple metrics, over the three ranges of positive to negative samples (1+:1-, 1+:2-, 1+:10-) (Figure 2). Spatial block cross-validation was not implemented due to data limitations but is recommended for future validation to better account for spatial dependence. “The model was retrained using the most relevant features selected in the previous step, and the performance was re-evaluated. Utilizing the trained model, predictions were made for 2D grid points and visualized as a risk map, which is a type of thematic map where areas are colored or patterned in relation to data variables. This allows for a spatial understanding of the risk of HPAI outbreaks. Finally, the importance of each feature was calculated and visualized as a bar graph. This can provide insights into which attributes are most influential in the prediction of HPAI outbreaks.

Spatial relationships were partially captured through distance-based and buffer-based features (e.g., proximity to outbreaks and wild bird nests, and counts within multiple spatial ranges), which allow the model to reflect localized clustering and proximity-driven risk patterns. However, explicit spatial autocorrelation models (e.g., spatial lag or spatial error models) were not implemented in this study. Similarly, temporal dependence was not explicitly modeled, as the analysis was based on aggregated outbreak data from 2005 to 2020 rather than time-resolved observations.

To enhance practical applicability, the risk map should be interpreted using decision-specific probability thresholds, rather than treating all areas above a fixed cutoff (e.g., 0.5) as equally high risk. For example, surveillance efforts may be prioritized in areas within the top quantiles of predicted risk. This approach reduces the likelihood of over-allocation of resources and mitigates concerns regarding false-positive interpretations of the model output. The 5 km grid resolution was selected as a balance between spatial detail and data availability, ensuring sufficient representation of environmental variability while avoiding excessive sparsity given the limited number of outbreak observations.

## 3 Results

### 3.1 Wild bird migratory routes and settlements

**Figure 3:**
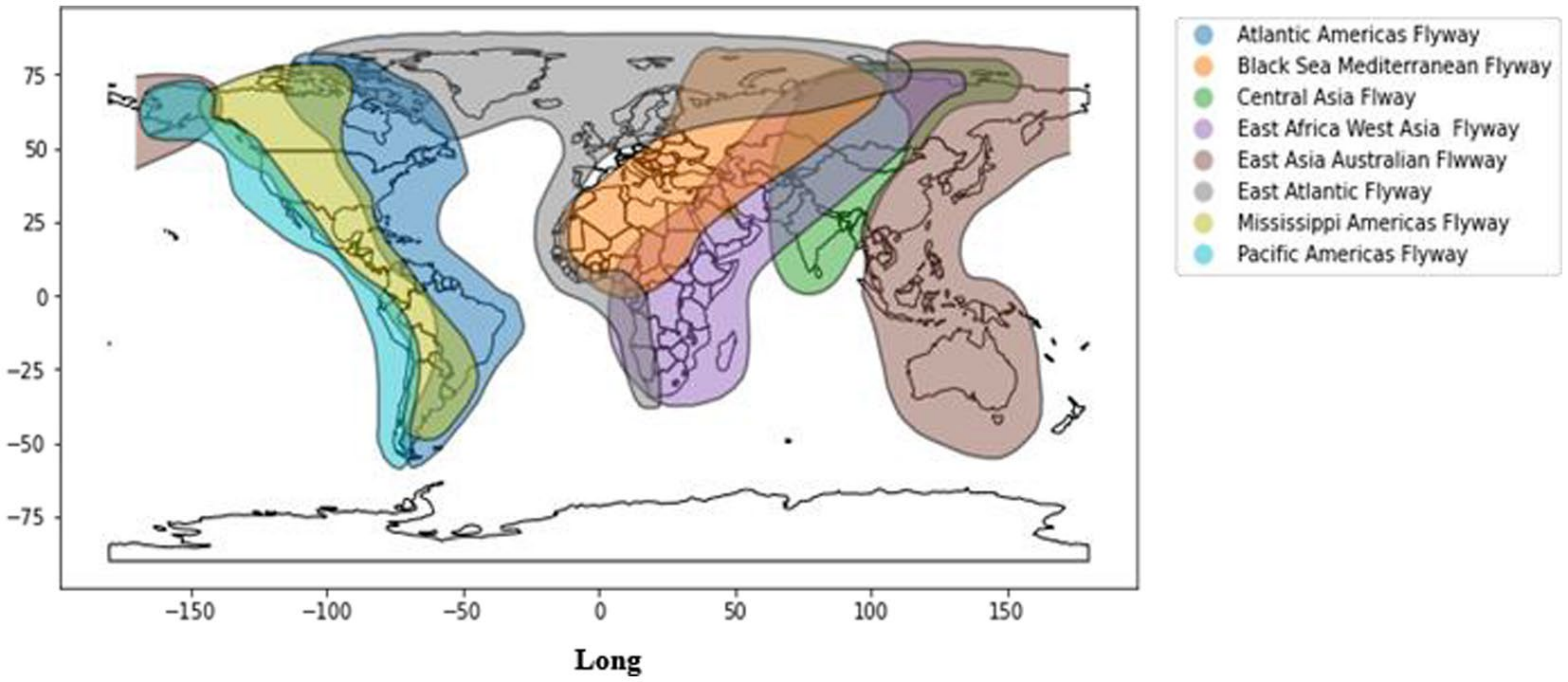
Map showing global migratory wild bird flyways.

**Figure 4:**
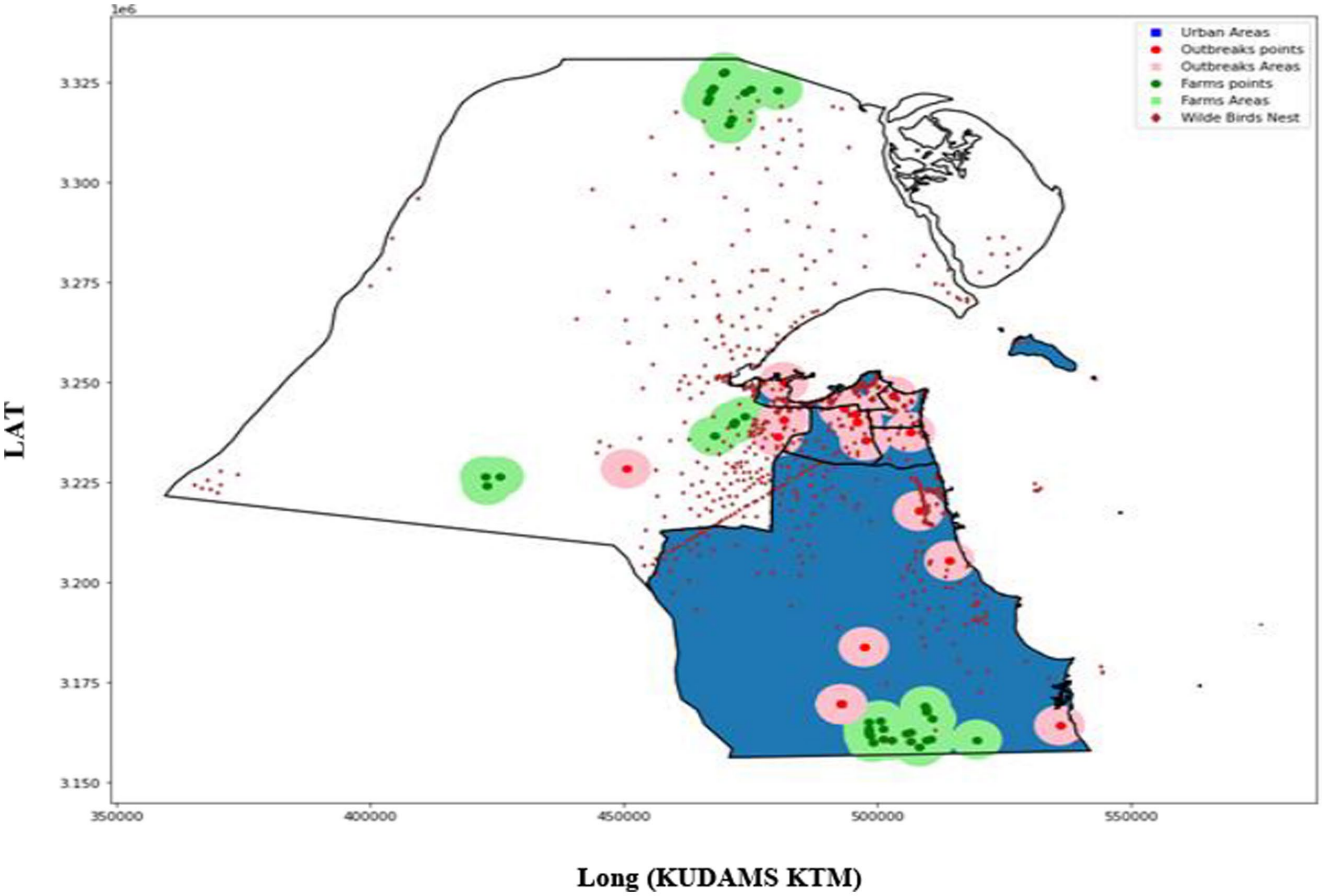
Kuwait map showing urban area, HPAI outbreak points and outbreak areas, farm points and farm areas, and wild bird nesting sites during the period 2005–2020.

It is important to understand the wild bird migratory routes and settlements in Kuwait since many wild birds carry the H5N1 virus, which can spread rapidly among flocks of domestic birds. HPAI spreads rapidly between farms through the movements of live birds, people, vehicles, and cages. Studies report more than 300 bird species in Kuwait; the majority are migratory species (18, 19). Kuwait is located within two primary migratory bird pathways: the Black Sea/Mediterranean flyway and the East Africa – West Asia flyway (Figure 3). Some of the locations where migratory birds were identified include Abdali and Wafra, north and south of Kuwait, which are located near major poultry farms (Figure 4). Tidal mudflats and sabkhas are characteristic ecosystems of Kuwait and attract a large number of migratory birds throughout the year. There are extensive mudflats, especially in the northern coastal area. The breeding population of Crab plover (*Dromas ardeola*) on Bubiyan island is probably the largest in the world, while Kubbar island is historically home to colonies of breeding seabirds, where thousands of bridled tern (*Onychoprion anaethetus*), white-cheeked tern (*Sterna repressa*) and hundreds of lesser crested tern (*Thalasseus bengalensis*) breed annually.

### 3.2 Pseudo-absences

**Figure 5:**
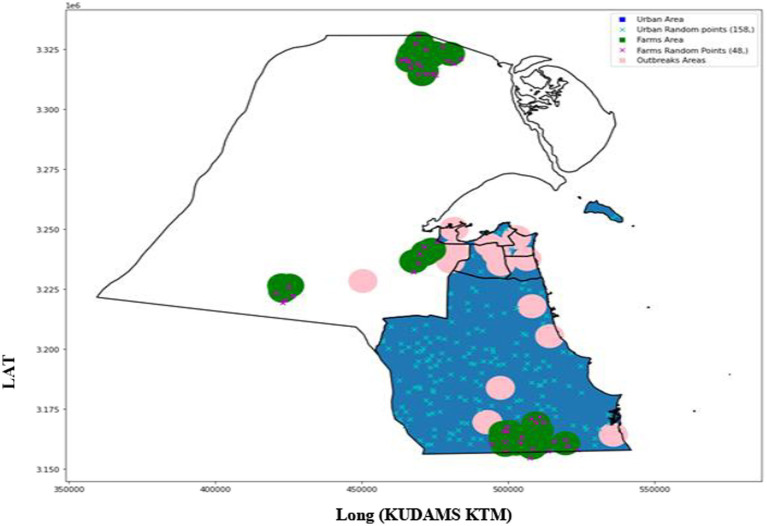
Kuwait map showing urban random points (158 points) and farm random points (48 points) used in the Logit model.

**Figure 6:**
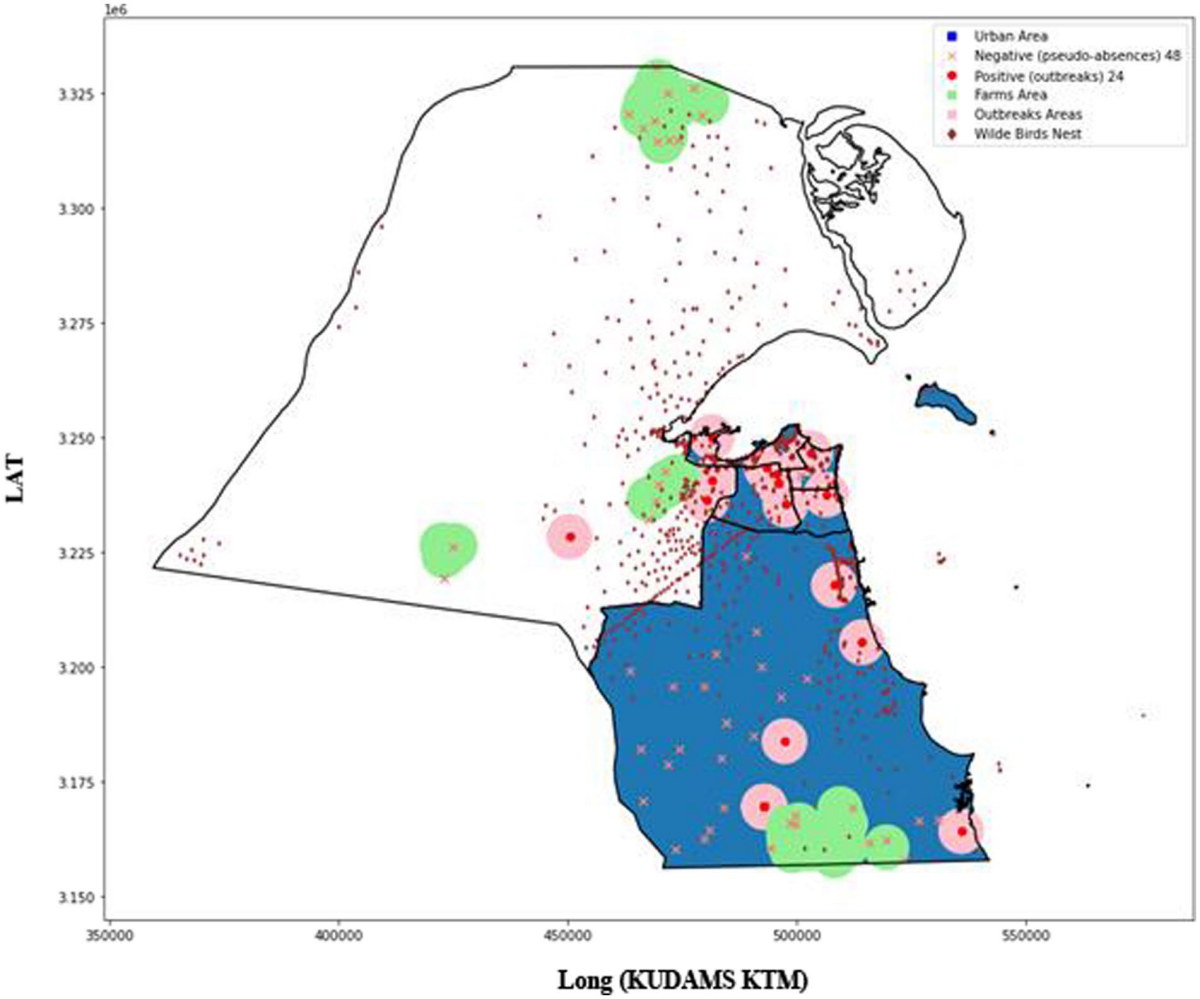
Kuwait map showing actual HPAI outbreak points (24 points) and negative (pseudo-absences) (48 points) along with wild bird nesting locations.

To predict the risk of an HPAI outbreak, we used logistic regression modeling (Logit). The Logit model used HPAI outbreak locations as positive cases and non-outbreak locations as negative cases. Negative cases were determined as any location that did not record an HPAI outbreak and was outside a buffer zone of 5 km from a recorded HPAI outbreak. Point locations were generated randomly; 50% in urban areas (24 of 158 urban random points) (blue zone) and 50% in farm areas (green zone) (24 of 48 farm random points) (Figure 5). Seven features were calculated for each positive and negative point location and used as inputs in the model. The seven features consisted of one distance feature and six buffer features. The distance was calculated based on the distance from the point to the nearest wild nesting site containing more than 100 birds. The buffers were calculated as counts of the number of wild bird nests (three buffers) and counts of the number of HPAI outbreaks (three buffers) with each buffer at a range of 10 km, 20 km, and 30 km from each location point (Figure 6).

### 3.3 HPAI risk model buildup

Logistic regression was used to predict the probability of an HPAI outbreak using Python statsmodels. The seven features were used as inputs in the model (predictor variables) and the HPAI outbreak was the predicted outcome (dependent variable). The Logit fit function performs a best fit between the predictors and the dependent variable. Model accuracy was calculated using multiple metrics functions from the scikit-learn library.

The best performing model included five features: (a) Outbreak within 10 km, (b) Distance-to-nearest wild nest (km), (c) Distance-to-nearest outbreak (km), (d) relative humidity (%) (e) Rainfall (mm). Its balanced accuracy of 0.79 and a high precision of 0.95 showed that it is reliable when predicting an outbreak and effectively handles imbalanced data. However, the recall score of 0.60 suggests that it missed a significant number of actual outbreaks. The model’s overall performance is strong, as indicated by its f1 score of 0.79 and an excellent ROC AUC score of 0.83, which confirms its high capacity to distinguish between outbreak and non-outbreak locations. The low negative log loss of −0.4309 and quick fit and score times indicate that the model is both accurate and computationally efficient.

**Figure 7:**
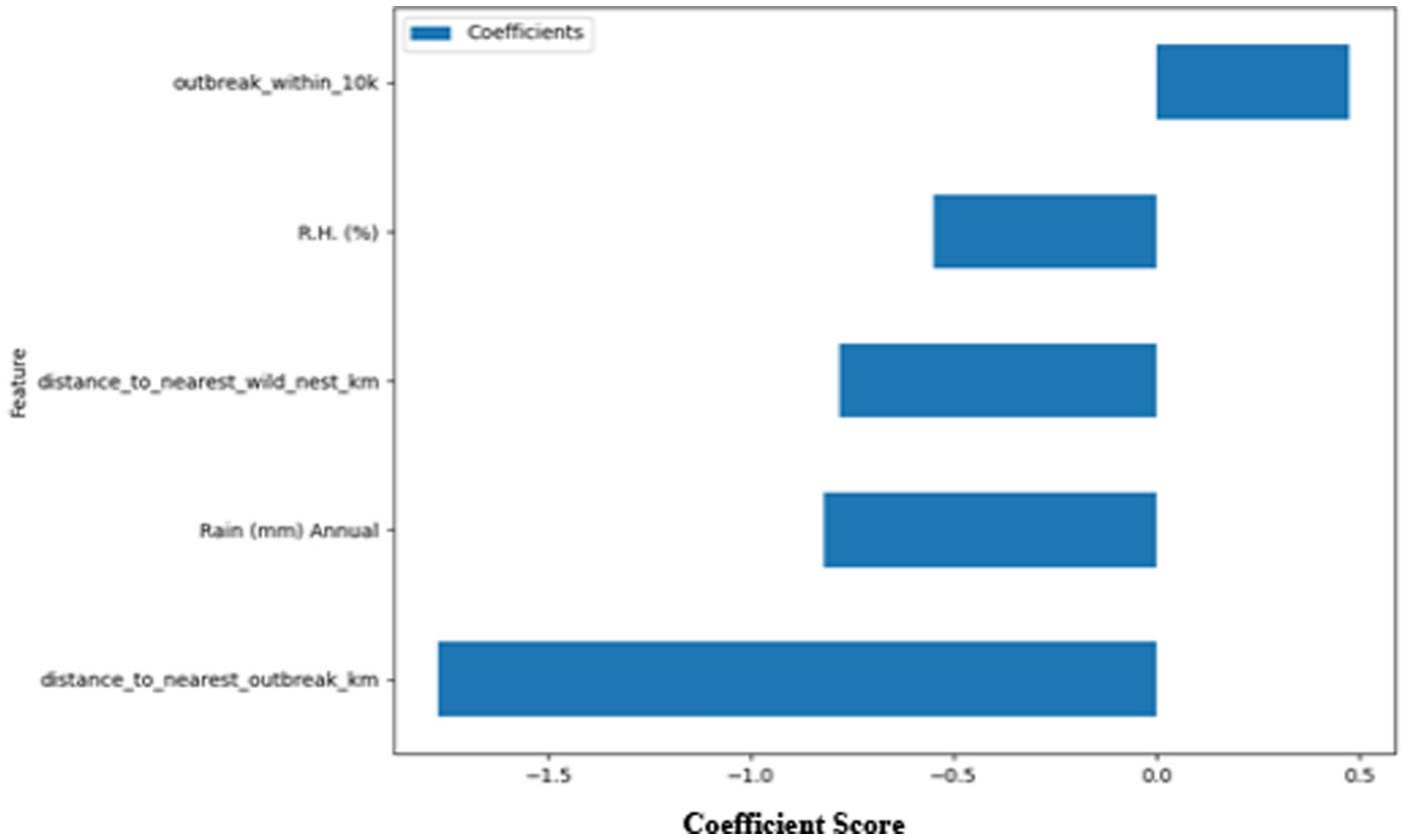
Feature importance for the prediction of HPAI outbreak in Kuwait.

The coefficients from the logistic regression model explained how each factor contributed to the likelihood of an avian influenza outbreak. A positive coefficient, such as the one for outbreak within 10 km, indicated that having more past outbreaks nearby increases the probability of a new outbreak. Conversely, negative coefficients, like those for distance-to-nearest outbreak (km), distance-to-nearest wild nest (km), RH (%), and Rain (mm), suggest that being farther from past outbreaks and wild nests, and having higher humidity and rainfall, all decrease the risk of an outbreak. The model predicts the probability of an outbreak at a given location by combining the values of its features with these coefficients; a higher combined score results in a greater predicted probability of an outbreak (Figure 7).

### 3.4 HPAI risk map

**Figure 8:**
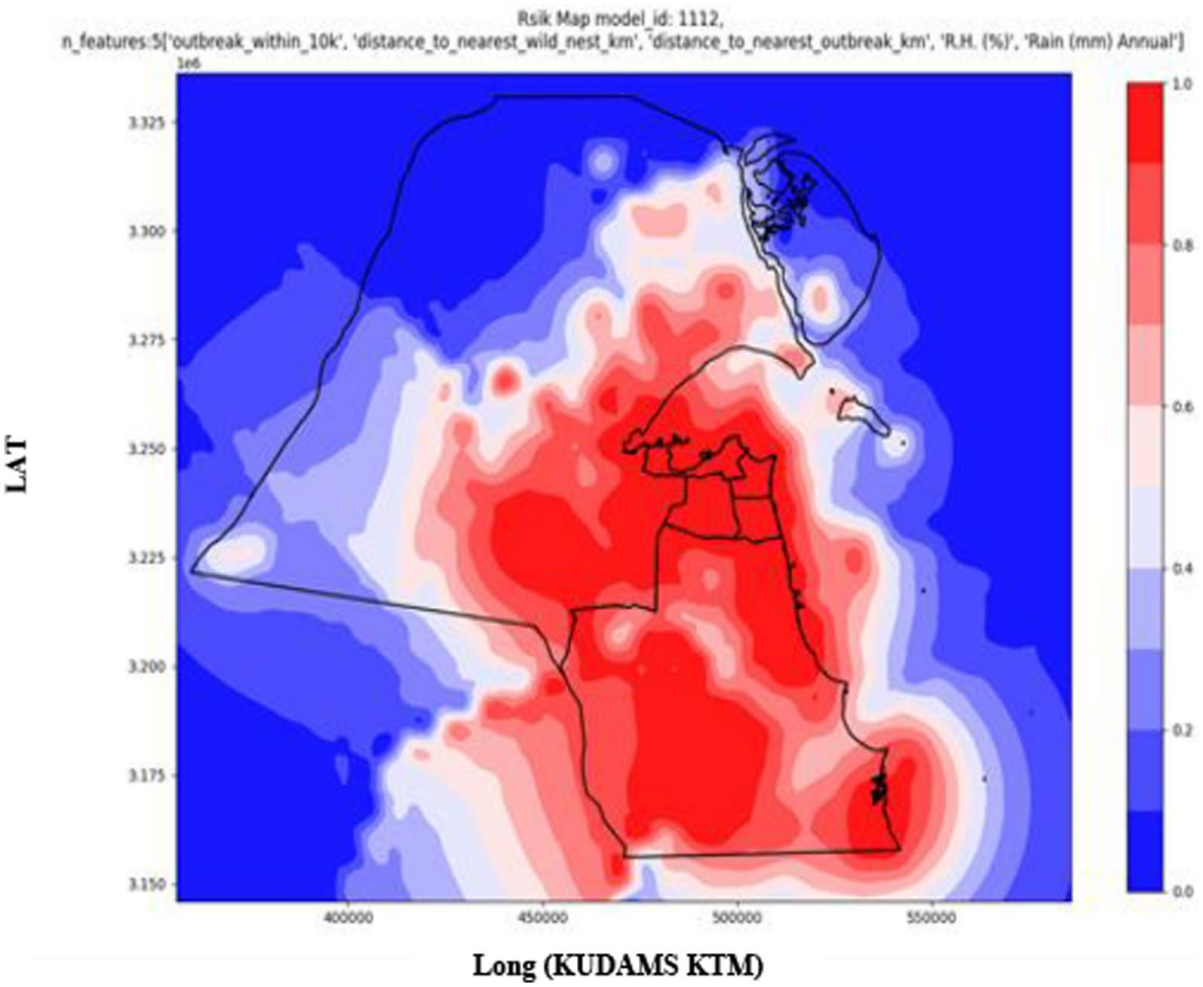
HPAI risk map showing predicted probability of outbreak across Kuwait (red: relatively high predicted probability, blue: low probability, white: intermediate uncertainty).

To produce the HPAI risk map, we generated uniform grid points at various distances across Kuwait (5, 10, 20, 30, 100 km). At each grid point, we calculated the seven features mentioned previously (1 distance, 3 HPAI outbreak buffers, 3 wild bird nest buffers). We used all grids as inputs into the model and calculated a probability value for each grid point. We interpolated the probability values using the Matplotlib triangulation function to produce the risk map. The probability values for each grid point range between 0 and 1. A risk map was generated by applying the best logistic regression model to a grid of points, with the probability of an outbreak at each point determining its color. Areas with high outbreak probability are shown in red, indicating “hotspots” that require targeted surveillance and prevention efforts, while areas in blue signify low risk. The light color at 0.5 indicates “uncertain” risk. The risk map showed that Kuwait metropolitan City and the seashore that extends from Kuwait center (Al-Joun) southward are the most vulnerable locations for HPAI outbreaks (Figure 8). Two other high-risk locations are also present to the west and southwest in Kuwaiti desert. The map represents a continuous probability surface and should be interpreted for risk prioritization rather than as a binary classification.

## 4 Discussion

This study identified Kuwait metropolitan City and the coastline extending southward from Al-Joun as the most vulnerable locations for HPAI outbreaks, consistent with their proximity to migratory bird pathways and the extensive mudflats and tidal sabkhas that attract large numbers of migratory birds year-round. These high-risk areas overlap with zones of significant poultry activity and human density, underscoring the importance of targeted surveillance and biosecurity enforcement in these locations.

Kuwait’s poultry sector is particularly exposed to disease introduction. Al-Nasser et al. (20) noted that high per capita poultry consumption (64.4 kg/cap/yr) necessitates heavy reliance on imports, while Al-Khalaifah et al. (5) documented how the SARS-CoV-2 crisis disrupted biosecurity infrastructure by restricting employee movement, interrupting trade, and increasing vaccination costs. Together, these vulnerabilities reinforce the need for spatially explicit risk tools that can guide resource allocation during both routine surveillance and crisis periods.

The logistic regression model achieved a balanced accuracy of 0.79 and ROC AUC of 0.83, demonstrating reasonable discriminative capacity for identifying outbreak-prone locations. However, several limitations warrant discussion. First, the recall of 0.60 indicates that the model missed approximately 40% of actual outbreaks—a significant shortcoming for an early warning application, where sensitivity is arguably more important than precision. Second, with only 24 positive outbreak locations over 16 years, the ratio of events to predictor variables is low, raising concerns about overfitting despite 5-fold cross-validation. Future iterations should consider penalized regression approaches or leave-one-out cross-validation to better assess generalizability. Third, the pseudo-absence design assumes that locations without reported outbreaks are true negatives, which may not hold where surveillance coverage is uneven. Fourth, the model was evaluated retrospectively on the same time period used for training; temporal validation—for example, training on 2005–2016 data and testing on 2017–2020 outbreaks—would more rigorously test the model’s predictive utility. A key limitation of this study is the lack of explicit modeling of spatial autocorrelation and temporal dependence. Although spatial structure was partially captured through engineered predictors such as distance to outbreaks and buffer-based counts, the model does not include formal spatial autocorrelation terms (e.g., spatial lag or spatial error components). In addition, the use of aggregated data over a 16-year period does not account for seasonal or interannual variation in HPAI outbreaks. These simplifications may limit the model’s ability to fully represent the spatiotemporal dynamics of disease spread. Future research should adopt spatiotemporal modeling approaches, such as Bayesian hierarchical models or geographically weighted regression, and incorporate time-stamped outbreak data to improve predictive performance and epidemiological interpretability.

A related methodological concern is the potential for spatial leakage arising from the inclusion of outbreak proximity variables (e.g., distance to the nearest outbreak and counts within 10 km, 20 km, and 30 km) in combination with conventional random 5-fold cross-validation. Because geographically proximate observations are likely to be spatially dependent, this evaluation strategy may result in training and validation sets that are not fully independent, thereby inflating performance metrics such as ROC-AUC and balanced accuracy. Furthermore, the use of proximity-based predictors derived from the same outbreak archive used for model training and validation introduces a risk of information leakage, particularly when such variables implicitly encode contemporaneous outbreak patterns.

To improve robustness, future work should adopt spatially explicit validation approaches, such as spatial block cross-validation or leave-location-cluster-out validation, which better account for spatial dependence and provide a more realistic estimate of out-of-sample predictive performance. Spatial block cross-validation was not implemented in the current study due to data constraints. In addition, sensitivity analyses should be conducted by excluding proximity-based predictors or incorporating temporally lagged outbreak variables to better reflect a true forecasting setting. While such approaches may reduce apparent model performance, they will yield more conservative and operationally meaningful estimates of predictive ability for early warning applications. Future research should also adopt spatiotemporal modeling approaches, such as Bayesian hierarchical models or geographically weighted regression, and incorporate time-resolved outbreak data to improve predictive performance and epidemiological interpretability.

These limitations may affect both the interpretability and the predictive validity of the model. In particular, the absence of a temporally explicit structure, combined with conventional cross-validation, may lead to optimistic estimates of model performance and does not fully reflect real-world forecasting conditions. To address these issues, future research should adopt a spatiotemporal modeling framework that incorporates time-resolved outbreak data. This would include structuring the dataset into year-wise or season-wise observations, integrating lagged covariates (e.g., prior outbreak occurrence and antecedent meteorological conditions), and implementing out-of-time validation strategies, such as training on earlier years and testing on temporally independent later periods. Such approaches would reduce the risk of temporal information leakage, improve the robustness of model evaluation, and enable a clearer distinction between retrospective explanation and forward-looking risk prediction. Additionally, the integration of advanced spatiotemporal methods, such as Bayesian hierarchical models or geographically weighted regression, would further enhance the model’s ability to capture the complex dynamics of HPAI transmission.

Given the relatively small number of positive outbreak events (*n* = 24) in relation to the number of predictors retained in the final model, the events-per-variable ratio is limited, which may increase the risk of overfitting and reduce the stability of coefficient estimates. In the present study, model interpretation is based primarily on point estimates, and a more comprehensive statistical characterization—including reporting of standard errors and confidence intervals—would provide a clearer quantification of uncertainty.

In addition, multicollinearity among predictors was not formally assessed using variance inflation factors (VIF), and model calibration was not explicitly evaluated. Future analyses should incorporate these diagnostics to improve model interpretability and assess the agreement between predicted probabilities and observed outcomes. Furthermore, the application of penalized logistic regression approaches, such as ridge or LASSO regularization, would be valuable for assessing coefficient stability and mitigating potential small-sample bias. Comparing results from standard and penalized models would strengthen confidence in the robustness and generalizability of the identified risk factors.

The feature set did not include poultry density, which multiple studies have identified as a key predictor. As described in Section 2.5, the predictor set was intentionally restricted to variables with spatially consistent coverage across the study period, which limited the inclusion of several anthropogenic and production-related factors such as poultry density, market locations, and movement networks. The feature set therefore did not include poultry density, despite its recognition in the literature as an important predictor of HPAI risk. This omission reflects constraints in the availability of harmonized, high-resolution datasets rather than a lack of epidemiological relevance. Consequently, the model primarily captures environmentally driven and proximity-based transmission processes, while human-mediated drivers of disease spread may be underrepresented. Future work should aim to incorporate these additional covariates to improve causal interpretability and reduce the risk of omitted variable bias. Schreuder et al. (21) found that wild bird density, particularly of waterbird species, significantly predicted HPAI outbreaks in the Netherlands beyond what land cover variables alone could explain. In the Kuwait context, Al-Nasser et al. (22) reported that 57.14% of specialized poultry farms are concentrated in Al-Wafra, with most operating at medium technology levels—a geographic concentration that, combined with varying biosecurity capacity, makes poultry density and farm-level characteristics important candidates for future model refinement.

The reliability of the risk surface depends partly on the density and spatial distribution of the 18 meteorological stations used for kriging interpolation. Leave-one-station-out cross-validation indicated reasonable accuracy for the four climate variables, but prediction uncertainty is inherently higher in areas distant from stations, particularly the southern desert and offshore islands. The bootstrap confidence surface revealed that the identified high-risk zones in metropolitan Kuwait and the coastline are robust to resampling variability, whereas risk estimates in data-sparse regions are more uncertain. Grid sensitivity analysis showed that the spatial pattern of high-risk areas was stable across 5 km, 10 km, and 20 km resolutions, suggesting that the main conclusions are not artifacts of the chosen grid spacing. Nevertheless, the kriging variogram was fitted assuming stationarity, which may not hold across Kuwait’s diverse coastal-to-desert gradient. Future work should explore non-stationary kriging or regression-kriging approaches that incorporate auxiliary covariates (e.g., elevation, distance to coast) to further reduce interpolation error.

Our approach complements existing surveillance tools for the region. Counotte et al. (23) demonstrated a generic risk assessment framework for HPAI entry through wild birds in the Netherlands, emphasizing transparent estimation with accessible data. Al-Hemoud et al. (7) developed an analytic web-based system using WOAH and WAHIS data to monitor animal disease outbreaks in Kuwait, identifying HPAI as the most frequently reported disease. Yoon et al. (24) validated a risk assessment program across 544 poultry farms in Korea, while Gonzales and Elbers (9) established farm-level reporting thresholds for HPAI detection in layer operations. Our spatial risk mapping extends these efforts by providing location-specific outbreak probability estimates tailored to Kuwait’s geography and ecology.

Future work should incorporate poultry density and farm biosecurity data, explore more flexible machine learning algorithms such as random forests and gradient boosting, and validate the risk map prospectively as new outbreak data become available. Such refinements would strengthen the model’s utility for informing both routine surveillance priorities and the siting of new poultry operations.

## 5 Conclusion

The study provides valuable insights into the wild bird migratory routes and settlements in Kuwait, which can be crucial for predicting the risk of HPAI outbreaks. The identification of high-risk areas can support authorities in implementing targeted surveillance and preventive measures to mitigate the spread of HPAI. Furthermore, incorporating additional environmental, epidemiological, and anthropogenic factors in future studies may enhance the accuracy and robustness of risk prediction models. The findings of this study also highlight the importance of promoting biosecurity practices among live bird traders and poultry market operators to effectively prevent and control HPAI outbreaks.

## Data Availability

The raw data supporting the conclusions of this article will be made available by the authors, without undue reservation.

## References

[1] Al-Khalaifah H Al-Nasser A. Cytokines as effective elements of the avian immune system. J Microbiol Genet. (2018) 3:2574–7371. doi: 10.29011/2574-7371.00019, 41802656

[2] Inam SA. A review of artificial intelligence for predicting climate driven infectious disease outbreaks to enhance global health resilience. Discover Public Health. (2025) 22:738. doi: 10.1186/s12982-025-01167-4

[3] Perez-Saez J King AA Rinaldo A Yunus M Faruque AS Pascual M. Climate-driven endemic cholera is modulated by human mobility in a megacity. Adv Water Resour. (2017) 108:367–76. doi: 10.1016/j.advwatres.2016.11.013, 29081572 PMC5654324

[4] Nusrat F Haque M Rollend D Christie G Akanda AS. A high-resolution earth observations and machine learning-based approach to forecast waterborne disease risk in post-disaster settings. Climate. (2022) 10:48. doi: 10.3390/cli10040048

[5] Al-Khalaifah H Al-Nasser A Abdulmalek N Al-Mansour H Ahmed A Ragheb G. Impact of SARS-con-V2 on the poultry industry in Kuwait: a case study. Front Vet Sci. (2020) 7:577178. doi: 10.3389/fvets.2020.577178, 33195579 PMC7536295

[6] Jebara B Karim M. WAHIS-wild and its interface: the OIE worldwide monitoring system for wild animal diseases. Vet Ital. (2016) 52:91–100. doi: 10.12834/VetIt.235.779.3, 27393871

[7] Al-Hemoud A AlSaraf M Malak M Al-Shatti M Al-Jarba M Othman A . Analytical and early detection system of infectious diseases and animal health status in Kuwait. Front Vet Sci. (2021) 8:676661. doi: 10.3389/fvets.2021.676661, 34395570 PMC8359926

[8] Al-Khalaifah H Alotaibi M Al-Nasser A. The relation between avian coronaviruses and SARS-CoV-2 coronavirus. Front Microbiol. (2022) 13:976462. doi: 10.3389/fmicb.2022.976462, 36312988 PMC9608149

[9] Gonzales JL Elbers AR. Effective thresholds for reporting suspicions and improve early detection of avian influenza outbreaks in layer chickens. Sci Rep. (2018) 8:8533. doi: 10.1038/s41598-018-26954-9, 29867092 PMC5986775

[10] Martin V Pfeiffer DU Zhou X Xiao X Prosser DJ Guo F . Spatial distribution and risk factors of highly pathogenic avian influenza (HPAI) H5N1 in China. PLoS Pathog. (2011) 7:e1001308. doi: 10.1371/journal.ppat.1001308, 21408202 PMC3048366

[11] Soares Magalhães RJ Ortiz-Pelaez A Thi KLL Dinh QH Otte J Pfeiffer DU. Associations between attributes of live poultry trade and HPAI H5N1 outbreaks: a descriptive and network analysis study in northern Vietnam. BMC Vet Res. (2010) 6:10. doi: 10.1186/1746-6148-6-10, 20175881 PMC2837645

[12] Yupiana Y de Vlas SJ Adnan NM Richardus JH. Risk factors of poultry outbreaks and human cases of H5N1 avian influenza virus infection in West Java Province, Indonesia. Int J Infect Dis. (2010) 14:e800–5. doi: 10.1016/j.ijid.2010.03.014, 20637674

[13] Al-Nasser A Al-Khalaifah H Al-Bahouh M Khalil F Ragheb G Ahmed A. "Poultry research achievements in Kuwait and way forward". In: Newest Updates in Agriculture and Veterinary Science, London, United Kingdom: BP International (2022) 1, 18–33.

[14] Phillips SJ Dudík M Elith J Graham CH Lehmann A Leathwick J . Sample selection bias and presence-only distribution models: implications for background and pseudo-absence data. Ecol Appl. (2009) 19:181–97. doi: 10.1890/07-2153.1, 19323182

[15] Hirzel AH Helfer V Metral F. Assessing habitat-suitability models with a virtual species. Ecol Model. (2001) 145:111–21. doi: 10.1016/S0304-3800(01)00396-9

[16] VanDerWal J Shoo LP Graham C Williams SE. Selecting pseudo-absence data for presence-only distribution modeling: how far should you stray from what you know? Ecol Model. (2009) 220:589–94. doi: 10.1016/j.ecolmodel.2008.11.010

[17] Barbet-Massin M Jiguet F Albert CH Thuiller W. Selecting pseudo-absences for species distribution models: how, where and how many? Methods Ecol Evol. (2012) 3:327–38. doi: 10.1111/j.2041-210x.2011.00172.x

[18] Al-Sirhan A. Al-Bathali O.. (2010). The Kuwait Bird Market January to May 2010. Available online at: http://www.oskonline.Org/reports/BirdMarket.Pdf (Accessed May 29, 2012)

[19] El-Gamily H Al-Damati T Fayad M Thomas B R H. Geographic Information system risk Analysis Application for Avian Influenza Outbreak in Kuwait. Kuwait: Kuwait Institute for Scientific Research. Final Report – KISR 9559. (2009).

[20] Al-Nasser A Al-Khalaifah H Khalil F Al-Mansour H. Poultry industry in the Gulf cooperation council with emphasis on Kuwait. Worlds Poult Sci J. (2020) 76:577–89. doi: 10.1080/00439339.2020.1782802

[21] Schreuder J de Knegt HJ Velkers FC Elbers AR Stahl J Slaterus R . Wild bird densities and landscape variables predict spatial patterns in HPAI outbreak risk across the Netherlands. Pathogens. (2022) 11:549. doi: 10.3390/pathogens11050549 35631070 PMC9143584

[22] Al-Nasser A Al-Khalaifah H Al-Mansour H Ahmad A Ragheb G. Evaluating farm size and technology use in poultry production in Kuwait. Worlds Poult Sci J. (2020) 76:365–80. doi: 10.1080/00439339.2020.1737625

[23] Counotte MJ Petie R Van Klink EG De Vos CJ. A generic risk assessment model for animal disease entry through wildlife: the example of highly pathogenic avian influenza and African swine fever in the Netherlands. Transbound Emerg Dis. (2023) 2023:9811141. doi: 10.1155/2023/9811141, 40303804 PMC12016805

[24] Yoon H Jang AR Jung C Ko H Lee KN Lee E. Risk assessment program of highly pathogenic avian influenza with deep learning algorithm. Osong Public Health Res Perspect. (2020) 11:239. doi: 10.24171/j.phrp.2020.11.4.13, 32864315 PMC7442435

